# Milk Quality and Animal Welfare as a Possible Marketing Lever for the Economic Development of Rural Areas in Southern Italy

**DOI:** 10.3390/ani11041059

**Published:** 2021-04-08

**Authors:** Lorenzo Infascelli, Raffaella Tudisco, Piera Iommelli, Fabian Capitanio

**Affiliations:** Dipartimento di Medicina Veterinaria e Produzioni Animali, Università di Napoli Federico II, 80100 Naples, Italy; lorenzo.infascelli@libero.it (L.I.); raffaella.tudisco@unina.it (R.T.); fabian.capitanio@unina.it (F.C.)

**Keywords:** milk quality, animal welfare, WTP

## Abstract

**Simple Summary:**

The growing interest of consumers in products that guarantee animal welfare and a high level of quality should represent the economic lever for small producers in the south of Italy, who mainly manage their farms on pasture or feeding animals with a high forage/concentrate ratio diet. In response to new consumers’ needs, the proposal on the market of these products can be advantageous for small breeders who may require a higher price for the products thus obtained. Our research emphasizes the fact that a greater awareness about the qualities of milk as well as animal welfare positively influence the likelihood of increased consumers’ willingness to pay.

**Abstract:**

The aim of the present work was to investigate the consumers’ willingness to pay (WTP) for dairy products obtained by grazing animals or fed with a high forage/concentrate ratio diet. To this aim, a survey was carried out on Italian consumers in the Campania Region and data collected were analyzed both by simple descriptive statistics and by an econometric model. Our results highlight that young age, knowledge of milk properties, and a healthy lifestyle are extremely important components in determining a higher WTP.

## 1. Introduction

European consumers pay great attention to food and agricultural products characterized by ethical and nutritional aspects linked to the welfare of food-producing animals [1]. In this context, EU citizens could consider labelling as a substitute of information about animal welfare. European policy makers foster a set of shared regulations on animal protection to tackle consumer attention for these aspects.

The origin of European legislation aimed at protecting animal welfare dates back to 1986 and at the beginning, it concerned only laying hens. Over the years, with the regulations of 1991 and 1998 (98/58/EC), interventions on protecting animal welfare have been extended to other species (calves, pigs, fish, reptiles, and amphibians) and several standards for transport, stunning, and slaughter have been adopted (http://ec.Europa.eu/food/animals/welfare/practice/farm/index_en.htm (accessed on 20 November 2019)).

The possibility of using labels on animal-derived food certifying them as obtained exceeding the minimum animal welfare standards has long been debated, but an official European label has not been proposed yet, due to the difficulty of dealing with a complex issue such as animal welfare [2].

Consumers’ needs are the force that should foster the development of the market toward a direction that is able to satisfy these needs. From this perspective, consumer choices can change production technologies and marketing strategies as they determine which products will be accepted or not by the market [3,4,5].

Consequently, product quality, taste, and healthiness should motivate paying higher prices for meat and dairy products from breeding systems with lower environmental impact and better animal welfare [6,7]. In addition, consumers appreciate landscape attributes such as the presence of grazing animals [8]. Grazing improves animal welfare [9], biodiversity, and cultural landscape conservation [10], preserving the attractiveness of life in rural areas [11,12]. Consumers associate animal welfare with product quality, taste, and healthiness [13].

For this aim, we structured a questionnaire aimed to investigate consumer preferences toward milk quality and animal welfare. Before carrying out the questionnaire, each interviewer explained, by reading a written sentence, what we investigating in our research in terms of the relationship between feeding diet, milk quality, and animal welfare. We emphasized that an appropriate feeding system of livestock guarantees the presence in the milk of several beneficial compounds such as polyunsaturated fatty acids, CLAs, and polyphenols, which are known to play a positive role in human health. In order to increase the quantity of such molecules in milk, it is important to set up a diet for the animals that includes the use of pasture or a diet with a high forage/concentrate ratio because they are able to guarantee an abundance of chemical precursors of the beneficial substances above-mentioned. Farms that adopt these feeding strategies also ensure animal welfare, since in this way, the animal is guaranteed the opportunity to express its natural behavior. Furthermore, grazing or, alternatively, a diet with a high forage/concentrate ratio, determines an improvement in the oxidative status of the animals (which can be considered among the factors that express animal welfare) mainly due to an increase in antioxidant compounds. Specifically, for this purpose, consumers were asked at the end of the questionnaire what percentage of the price they would be willing to pay both for having a milk produced by animals fed on pasture or with a high forage/concentrate ratio diet and for the well-being of extensively reared animals. The results collected by questionnaire were analyzed by an econometric analysis to investigate which consumer profile positively affected animal product purchase in the sense above-mentioned.

### 1.1. Milk Production in Rural Areas

In Mediterranean countries, a seasonal pasture scarcity exists, thus, particularly in summer and winter, farmers increase the concentrate feed supply often without a technical assessment of the real needs of the animals. Monitoring milk yield and animals’ body score could determine an improvement in the feeding management; nevertheless, a balance between the monitoring costs and benefits should be analyzed previously.

The impact of pasture seasonality could be reduced by:

- planning of livestock reproductive and feeding management throughout the synchronization of periods of highest animal nutritive requirements (end of pregnancy and lactation) with high pasture availability [14];
- a better use of Mediterranean woody fodder species for animal feeding when herbaceous pasture is scarce (summer);
- growing fodder crops on the farm in the winter and summer. This strategy has low feasibility in areas where steep terrain and shallow soils hinder land cultivation [15] as in mountains where it would be better to improve pasture yield and quality by oversowing every few years with legumes or other selected species, if expertise and machine equipment are available;
- short-distance livestock mobility [16], even if it may have a negative impact on wildfire prevention success.

On the other hand, raising livestock on pastures does not appear to be attractive: very long working hours and low pay for shepherds. This results in a progressive decrease in grazing systems with marginal areas abandoned [17], which causes an accumulation of biomass and the expansion of shrublands and forestlands [18]. In Mediterranean countries, due to the long hot and dry season, this implies a higher risk of wildfires.

Regardless, the consumer approval for an improvement of animal welfare, biodiversity and cultural landscape conservation, and high quality of products could be a great opportunity for livestock producers to sell pasture-raised products at higher prices. This multifunctionality of extensive livestock should receive also greater recognition and be better remunerated through public support.

### 1.2. Milk Quality and Animal Welfare

Our research group carried out several trials aimed at evaluating the influence of feeding on the milk nutritional aspects [19,20,21,22,23,24,25] as well as on animal welfare [26,27]. Particular interest was focused on the milk fatty acid profile, among the others in the essential fatty acids n-3 and n-6, two classes with opposite physiological functions (i.e., pro- and anti-inflammatory activity for n-6 and n-3, respectively), and on the conjugated linoleic acids (CLAs). Several studies have demonstrated the beneficial effects of n-3 fatty acids in the prevention of coronary heart disease, hypertension, type 2 diabetes, rheumatoid arthritis, and some other diseases [28]. A n-6:n-3 ratio ranging from 2 to 4 is considered optimal for human health [29]. Milk with low n-6:n-3 ratio and high CLA content positively affect the inflammatory state, oxidative stress, and mitochondrial function in rats [30,31].

Very recently [32], we carried out a trial aimed to estimate the effect of pasture on miRNA 103 expression of milk. The effects of such RNA molecules on the health of young and adult milk consumers represent a relevant aspect to explore. Indeed, a growing body of evidence indicates that microRNAs play a relevant role in the regulation of lipid metabolism-related genes [33]. This represents a burgeoning area of investigation as these molecules can represent a novel class of tools for cardiovascular disease (CVD) therapeutic intervention.

Concerning animal welfare, feeding with a high forage/concentrate diet results in an improvement of animal oxidative status, mainly due to an increase in antioxidant com-pounds [26] that may prevent mastitis and a decrease in milk production [34].

### 1.3. Adding Value to Agri-Food Products

We have above emphasized the milk nutritional quality due to the feeding system and the effect both on landscape and animal welfare; a crucial aspect would be played by the possibility of consumers recognizing these attributes.

The benefits derived from the certification of products contribute to repaying the individual producer for the costs incurred. These benefits include the possibility of selling the product at a premium price; defense against unfair competition; the use of the label obtained through certification as a differentiation tool; the stabilization of commercial relationships; the development of new channels and markets; the possibility of using collective marketing; and the guarantee to consumers regarding the local origin, traditional methods and quality [35,36,37]. More generally, the adhesion to a label by an agri-food enterprise ensures the recognition of the product in the market and allows for a series of unquestionably advantageous results to occur in every phase of the value chain, from primary production to transformation.

The Product Specification (PSs) such as Protected Designation of Origin (PDOs), Protected Geographic Indication (PGIs), and Traditional Specialty Guaranteed (TSGs) provide consumers with specific information on each product’s conformity to a system of rules determining its quality, thereby signaling the product’s credence attributes and diminishing consumer transaction costs from asymmetric information. This enhancement allows the promotion of quality products with defined characteristics, the diversification of agricultural production, fair, competitive conditions between branded products, greater competitiveness (differentiation strategy), and commercial advantages (exclusive use of the GI denomination) compared to standard products.

These issues should foster a new model of supply aimed to answer the consumer need in terms of a clear and transparent labelling of animal-friendly and quality products [38].

As above introduced, we found that an appropriate feeding system of livestock guarantees the presence in the milk of several beneficial compounds such as polyunsaturated fatty acids, CLAs, and polyphenols, explaining that in order to increase the quantity of such molecules in milk, it is important to set up a diet for the animals that includes the use of pasture or a diet with a high forage/concentrate ratio. Farmers that adopt these feeding strategies also ensure animal welfare, since in this way, the animal is guaranteed the opportunity to express its natural behavior. These aspects represent the starting point of our empirical analysis, which aimed to verify the degree of consumer interest in these qualitative attributes, in terms of willingness to pay. One of the aims of this paper was to investigate consumer knowledge with respect to the milk nutritional quality due to feeding strategies, and on the motivations that should foster the purchase or not of these products and the evaluation of the main consumer profile that would increase WTP for quality milk and animal welfare products.

We carried out a survey on Italian consumers in the Campania Region; data were processed both by descriptive statistics and by using an econometric model.

Specifically, the analysis efforts were directed toward two objectives: the first was the identification of emerging consumer profiles by a cluster analysis (CA) aimed to identify conceptual categories (consumer groups) that could merge the main profile of the interviewed toward both milk quality and animal welfare; the second one, by using a logit model, was carried out with the objective to explain the main consumer attributes that would increase the consumers’ WTP for milk quality and animal friendly products.

## 2. Materials and Methods

Aiming to investigate the consumer behavior, we carried out a questionnaire. We chose to use a semi-structured questionnaire, which started from more general questions to increase in more details at the end, placing those considered sensitive at the end. The questionnaire included 17 questions grouped into the eight sections below illustrated.

The information on welfare, nutrition, and its effects on milk quality with consequent health implications was provided concurrently before the administration of the questionnaire by the interviewer; the interviewers were students of the Science and Technology of Animal Production course who are familiar with the concepts of animal nutrition and animal welfare.

Specifically, the questionnaire was structured as follows:

Sec. 1—Shopping habits

Sec. 2—Sources of information to set the choices in purchasing food

Sec. 3—Knowledge report on diet-health

Sec. 4—Knowledge on milk quality attributes

Sec. 5—Motivation and knowledge to purchase “High forage milk”

Sec. 6—Lifestyle

Sec. 7—WTP “High forage milk” and animal welfare

Sec. 8—Socio-demographic

During the period between September and November 2020, a total of 218 questionnaires were administered in a face to face manner in the cities of Napoli, Salerno, and Caserta (Table 1).

### Cluster Analysis and Econometric Analysis

As a first step, we carried out a CA; the CA allowed us to divide the sample of interviewees into groups (or clusters) that were as homogeneous as possible within them and dissimilar to each other. The similarity and the difference are not the total, but refer to specific answer options that would reflect the most significant characteristics within each cluster. In our investigation, the division found to be more effective made it possible to highlight, within our sample of consumers, groups or clusters that will be illustrated in the following section and which allowed us to obtain a cross-section of the consumers interviewed by us.

We used CA to identify which factors had affected the consumers’ differentiations; specifically, we obtained an overview of the relationship between socio-economics, revenue, and education. We first verified these relationships by testing for differences between means and using the χ^2^ test, according to the quantitative nature, or otherwise, of the variables considered. Following this, we carried out a logistic regression to correlate characteristics from CA as well as some discrete indicators not included in the CA to the propensity of consumers to increase their willingness to pay for milk quality/animal health (yes/no).

For both CA and logistic regression, we used R software.

Specifically, the first group was made up of uninformed consumers on the subject of animal welfare and milk quality: they demonstrated that they did not have an awareness and knowledge of the subject.

Regarding the second cluster, it was made up of consumers interested in animal welfare and nutrition. They showed interest in animal welfare, and consequently, in animal friendly products.

The third cluster was represented by conscious consumers, who represented particular interviewees. They shared precise opinions, but these did not include those relating to purchasing behavior; hence, we can assume that they were interested in animal welfare, but linked to label presence.

The last cluster was made up of quality-conscious consumers. They had direct knowledge, at least influenced by advertising.

Overall, the latter cluster seems to include superficial consumers in their approach to animal welfare and related issues; furthermore, these respondents were not united by the purchasing behavior toward animal friendly products.

The step forward at CA was the implementation of the econometric analysis. To analyze the characteristics that positively influenced the willingness to pay (WTP) for quality, taste, and healthiness as well as identify which variables determine the significantly greater likelihood of a higher WTP, the respondent was provided with information on the feeding strategies and supplementary diet. After these specifications, the sample of respondents to the questionnaire were fully aware of the objectives of the food strategy administered to the animals and were able to express their degree of acceptability toward a type of product with certain qualities. Data obtained were utilized by a logistic regression model, in which the dependent variable used assumes the value of 1 if the consumer declares their willingness to pay more for these attributes with a level of certainty in response of at least 70%, and value 0 otherwise.

The formal specification of the model used is as follows

(1) $$P_{i}=P\left(Y_{i}=1\left|X_{i}\right.\right)=E\left(Y=1\left|X_{i}\right.\right)=\frac{1}{1+e^{-\left(\alpha +\beta X_{i}\right)}}=\frac{1}{1+e^{-Z_{i}}}$$

aiming to have a probabilistic evidence of the impact of independent variables on the likelihood that a consumer is available to have a higher WTP for such product. As in standard regression models, *α* is the intercept and *β* is the vector of coefficients for the vector of independent variables. The last part of the equation

(2) $$P_{i}=\frac{1}{1+e^{-Z_{i}}}$$

represents the logistic distribution, which takes a value between 0 and 1; this possibility warrants that each prediction on *X_i_*, the value assumed for *P_i_*, can be interpreted as a probability. This is one of the main reasons why a logit model was implemented for this analysis.

In order to estimate the Equation (2) must be rewritten so that it is linear in and *β*. Therefore, in order to estimate, the logit model was specified as:

(3) $$\ln\left(\frac{P_{i}}{1-P_{i}}\right)=\alpha +\beta X_{i}+\varepsilon_{i}$$

In Equation (3), the dependent variable represents the logarithm of the ratio of frequency of consumption of quality milk, whereas *ε_i_* represents the stochastic disturbance term. We could consider the estimated parameters as a change in the frequency of the likelihood that a consumer will buy quality milk and/or animal friendly products. Positive values imply that the growth of the variable *X_i_* will increase the likelihood that the respondent purchasing the products considered. In this work, negative values imply the opposite [39].

Aiming to estimate which variables could be included in the model, we used the likelihood ratio test. The null hypothesis is rejected if the LR test is greater than the value of the Chi-square (x2-value) with degrees of freedom equal to the number of independent variables used in the model.

In the logit model implemented in this analysis, aiming to estimate how the socio-economic factors characterizing the sample interviewed influence the WTP toward milk quality and animal friendly products, were used as independent variables the place of purchase, purchase frequency, age and the degree of education, attention to nutritional value, price and experience of consumption declared, buying habits, and finally, the degree of knowledge on milk quality and animal friendly products.

## 3. Results and Discussion

### 3.1. The Results of Exploratory Analysis

The application of CA allowed us to identify four homogenous groups of consumers (clusters) defined by the variables considered most representative in the explanation of the phenomenon investigated.

The sampling procedure was probabilistic with non-rational choice, based on a sample of people aged between 20 and 80 years, responsible for food spending for the family of reference (Table 2), distributed in the cities considered to be representative of the Campania Region (97 questionnaires administered in the city of Naples, 67 in Salerno, 54 in Caserta). Interviews were conducted in the vicinity of outlets of the big retail chains (GDO). Table 2 summarizes the main characteristics of the sample.

The first group represents 27% of the total and consists of the “uninformed consumers”. These people mainly belonged to the 50–64 age group (49%), characterized by a low-average level of education and the prevalence of low-middle income classes. The main characters took on a strong cluster homogeneity relative to the size of knowledge, in particular, it is consumers who claim not to know that any type of milk is linked to feeding strategies, who have never bought such products, and will probably continue to avoid these products in the future. They were also uninformed on the issues specifically related to food, for which there seems to be awareness of the food–health relationship. Ultimately, it is a consumption profile for which information and awareness related to food consumption takes a marginal character in the formation of preferences, and it is most likely engaged in routine consumer purchasing specifications.

The second cluster, which was 16% of the total sample, concerns the “consumers concerned about animal welfare”. Within the group fell respondents with the highest average age (50 years), characterized by a high degree and in the middle income classes. These consumers seemed to know the property linked to feeding strategies, which they have bought and will continue to do so.

The third was identified related to the consumption profile “conscious consumers”, which represents 25% of the sample. Consumers who fell in this group had a mean age of 40 years in the household, the prevailing level of education was high (62% graduated), and the income was medium-high. In this group, the respondents were firmly convinced of the real benefits to health from milk obtained by animals fed a high forage diet, but, nevertheless, the purchase of such products was incidental and only covered those with a label. In terms of attention to animal welfare, they showed strong interest on this aspect linked to environmental conservation.

The final group includes the “quality-conscious consumers” and comprised 32% of respondents. Families of reference of respondents were on average several (the largest between groups) and were characterized by a relatively greater number of younger people than that of the elderly. The profile was low-middle income, while the average educational level was high. The level of knowledge shown on milk obtained by animal fed high forage diet was greatly influenced by advertising campaigns; members of the group knew one of the most popular products with health claims and claimed to have made purchased milk driven by curiosity aroused by publicity.

In the following tables, the different profiles are described with reference to the most important socio-demographic variables compared to the overall distribution of the sample (Table 3, Table 4 and Table 5).

We can conclude from the cluster analysis that the consumers interviewed, for the most part, paid a lot of attention to the issue of animal welfare and the quality of milk linked to animal feeding. However, it is clear that the certification and labeling process also substantially influences the purchase act of several consumers.

### 3.2. The Results of the Econometric Analysis

The analysis for milk was replicated for animal welfare. The reason for this lies in the eventual possibility of the emergence, as suggested by other empirical studies [40], of finding similar consumer attributes among these two product categories.

The evidence derived from the logit model used to identify the dimensions that influence the WTP on milk quality property are presented in Table 6.

The analysis showed the relevance of the variables “knowledge of milk property”, “young age”, “lifestyle”, and “buy in supermarkets” compared with a higher probability of declaring a higher WTP and the relationship between higher income and greater WTP was negative. This eventuality underlines the fact that a greater awareness about the qualities of milk, the identification of those with particular lifestyles and the pursuit of a healthy life, they bring to the background the concerns about price and availability of income. These results are consistent with the findings in other studies, indicating that young age, knowledge of milk property, and a healthy lifestyle are key elements in determining a higher WTP [41]. Specifically, Maynard and Franklin [42] showed that WTP for milk products enriched with CLA was positively affected by the knowledge of nutritional principles, the perception of food–health relationship, and adopting a healthy lifestyle.

The result achieved by the application of the econometric model to data on the milk quality aspect is as follows:

WTPMILK = − cost − β1Revenue class + β2Younger age + β3Lifestyle + β4Knowledge + β5Supermarket.(4)

The results show a similarity between the variables identified here and those previously noted. That is, that animal welfare as well as for milk positively influenced the likelihood of increased consumer WTP declared by the variables “knowledge of milk attributes”, “younger age”, “lifestyle” (with the combined presence of physical activity and dietary change); as expected, “buy in supermarkets” was not statistically significant. Thus, a negative connection emerges with the income class of membership for those who have expressed a willingness to a higher WTP for the purchase of milk. Otherwise, it indicates a strong influence of aspects linked to healthy lifestyle, but also of those with higher education.

WTPANIMAL W = − cost − β1Revenue class + β2Younger age + β3Lifestyle + β4Knowledge + β5Supermarket + β6 Higher education.(5)

## 4. Conclusions

The main objective of this work was to understand if consumers were amenable in rewarding an alternative method of animal nutrition which, from our point of view, has two substantial impacts: (1) improves the quality of the product (milk) and (2) increases animal welfare (as well as environmental factors).

Farms that adopt extensive breeding systems are able to ensure animal welfare as they guarantee adequate living conditions. Furthermore, this should translate into a better quality of the product obtained as a consequence of the link that exists between animal well-being and quality of product.

We emphasize the crucial role that both information and knowledge play in the dynamics of the market and what actually affects the demand for products aiming to enhance consumer WTP for taste and animal welfare. For this purpose, we informed those interviewed on the effects of feeding diet on both milk quality and animal welfare.

Based on the interviewed answers, as a first step, we carried out a CA with the aim to group the main consumer profile based on different attributes. We found four different groups: the first group demonstrated no awareness and knowledge of the subject. Regarding the second cluster, this was made up of consumers interested in animal welfare and nutrition. The third cluster were conscious consumers who represented particular interviewees. The fourth cluster was made up of quality-conscious consumers who had direct knowledge, or were at least influenced by advertising.

After carrying out the econometrics analysis, we found that consumers with specific features, especially in terms of knowledge, education, and age, showed a quite diffused interest toward the issues of product quality and animal welfare. They were willing to reward the higher milk quality and animal welfare, as introduced before the administration of the questionnaire, with a higher price. These results, which are limited to a sample of Campanian consumers, foster a reflection on the future needs in this perspective. One of the key aspects would be to understand how to transfer information to consumers on the higher quality of products, especially considering that often the only vehicle of certification or brand does not adequately remunerate farmers.

As this methodology was based on the determination of consumer knowledge and WTP for milk obtained by animals fed a high forage diet and their quality attributes, considering the above-mentioned on product valorization and market information, it would be very interesting in future studies to focus on the point of view of the producers rather than the consumers.

Our investigation, nevertheless limited in sample size and stratification, emphasizes that both quality-fed milk and animal welfare could represent a new sustainable, competitive, and replicable development model for rural and mountain areas in EU countries, especially from the perspective of the European Green Deal Policy for the CAP post 2020. This kind of economic approach could overcome the concept of the mountains only as a place of lost naturalness, heritage, and as a not competitive economy, in opposition to urban areas. It is clear that a turning point for the valorization of animal welfare and quality product aiming to sustain economic resilience in rural areas will be crucially played by the capability to manage an efficient cooperation among farmers. The promotion of environmental and food education programs through the organization of educational farms is a new form of multifunctional agriculture, which makes the territory known and links the production of agricultural goods to the provision of services to people in rural areas. It is a form of agriculture that aims to re-establish the link between food and community, thus strengthening social networks around food production and generating environmental, economic, and social self-sustainability. Since awareness of quality has been found to be a relevant parameter in the WTP, it is important to start developing educational projects in order to inform consumers, not only through more understandable and detailed labels, but also through school projects and educational meetings open to the public. Moreover, among other aspects, the context in which these farms are managed has to be considered. Problems such as accessibility to transport and the quality of local public institutions (bureaucracy) are often among the main obstacles to the development of farms in rural areas, therefore, the removal of such impediments must be pursued in order to reduce their isolation.

## Figures and Tables

**Table 1:** Survey sample.

Characteristics	
Target	Campania—Regions
Field	Makers in the household food expenditure
Sample	218 interviews
Timing	2 September–30 November 2020

**Table 2:** Main characteristics of the sample.

Sample		
Age group		
	20–34	29%
	35–49	33%
	50–64	28%
	65–80	10%
Education level		
	Primary school	2%
	Junior high school	6%
	Senior high school	45%
	University	47%

Source: Direct investigation.

**Table 3:** Main characteristics of groups and comparison with the sample mean.

Cluster	%	Age Mean	Household Size	% of Family with Child Younger than 10	% of Family with Person Holder than 65
Uninformed Consumers	27%	46	2.4	6%	21%
Consumers Concerned About Health	16%	50	2.9	18%	13%
Conscious Consumers	25%	41	2.7	20%	5%
Health-Conscious Consumers	32%	38	3.2	19%	12%
Total	100%	45	2.9	16%	12%

Source: direct investigation.

**Table 4:** Characterization of groups with regard to qualification and comparison with the sample mean.

Cluster	Primary School	Junior High School	Senior High School	University	Total
Uninformed Consumers	7%	10%	46%	37%	100%
Consumers Concerned About Health	-	5%	49%	46%	100%
Conscious Consumers	-	7%	31%	62%	100%
Health-Conscious Consumers	5%	8%	53%	34%	100%
Total	3%	7%	44%	46%	100%

Source: Direct investigation.

**Table 5:** Characterization of groups with respect to income class and comparison with the sample mean.

Cluster	Less than 10,000€	From 11,000 to 20,000€	From 11,000 to 35,000€	From 36,000 to 50,000€	From 51,000 to 75,000€	More than 75,000€
Uninformed Consumers	3%	18%	39%	20%	12%	3%
Consumers Concerned About Health	-	14%	64%	21%	-	-
Conscious Consumers	3%	24%	26%	31%	18%	1%
Health-Conscious Consumers	8%	19%	32%	28%	10%	5%
Total	5%	21%	38%	27%	12%	3%

Source: Direct investigation.

**Table 6:** Characterization of groups with regard to qualification and comparison with the sample mean.

**Logit Maximum Likelihood Estimation**			
**Variable**	**Coeff.**	**Std Error**	**T-Ratio (Prob)**
COST	−0.22792	0.13911	−1.9189 (0.052)
Revenue class	−0.41661	0.15605	−2.7945 (0.008)
Younger age	0.28763	0.13756	2.5892 (0.037)
Lifestyle	0.33822	0.14465	3.2822 (0.004)
Knowledge	0.32882	0.13288	3.2781 (0.008)
Supermarket	1.6772	0.33221	4.2271 (0.001)

Factor for marginal effect computations = 0.27289. Maximized value of the log-likelihood function = −139.4254. Akaike Information Criterion = −138.7245. Schwarz Bayesian Criterion = −146.4891. Hannan-Quinn Criterion = −151.5362. Goodness fit = 0.71220. Pesaran–Timmermann test statistic = 4.9134 (0.000). Pseudo-R-Squared = 0.11838. Applying the model to data for animal welfare are summarized in Table 7.

**Table 7:** Factors affecting positively Willingness to Pay (WTP) for animal welfare.

Logit Maximum Likelihood Estimation			
**Variable**	**Coeff.**	**Std Error**	**T-Ratio (Prob)**
COST	−0.67111	0.16335	−39722 (0.000)
Revenue class	−0.27565	0.14201	−18522 (0.053)
Younger age	0.31564	0.15633	1.8744 (0.048)
Lifestyle	1.3418	0.29722	4.1217 (0.015)
Knowledge	0.39844	0.18776	3.8325 (0.009)
Supermarket	0.03762	0.42133	0.9947 (0.094)
Higher education	0.65774	0.43897	4.7338 (0.003)

Factor for marginal effect computations = 0.38182. Maximized value of the log-likelihood function = −122.3728. Akaike Information Criterion = −138.7822. Schwarz Bayesian Criterion = −145.7892. Hannan-Quinn Criterion = −149.8873. Goodness fit = 0.69344. Pesaran–Timmermann test statistic = 4.6755 (0.000). Pseudo-R-Squared = 0.12788.

## Data Availability

Not applicable.
